# The tree that hides the forest: cryptic diversity and phylogenetic relationships in the Palaearctic vector Obsoletus/Scoticus Complex (Diptera: Ceratopogonidae) at the European level

**DOI:** 10.1186/s13071-020-04114-1

**Published:** 2020-05-20

**Authors:** Antoine Mignotte, Claire Garros, Laetitia Gardès, Thomas Balenghien, Maxime Duhayon, Ignace Rakotoarivony, Laura Tabourin, Léa Poujol, Bruno Mathieu, Adolfo Ibañez-Justicia, Ahmet Deniz, Aleksandar Cvetkovikj, Bethan V. Purse, David W. Ramilo, Despoina Stougiou, Doreen Werner, Dubravka Pudar, Dušan Petrić, Eva Veronesi, Frans Jacobs, Helge Kampen, Isabel Pereira da Fonseca, Javier Lucientes, Javier Navarro, Josue Martinez de la Puente, Jovana Stefanovska, Kate R. Searle, Khalid Khallaayoune, C. Lorna Culverwell, Magdalena Larska, Maria Bourquia, Maria Goffredo, Marina Bisia, Marion England, Matthew Robin, Michela Quaglia, Miguel Ángel Miranda-Chueca, René Bødker, Rosa Estrada-Peña, Simon Carpenter, Simona Tchakarova, Sofia Boutsini, Ståle Sviland, Stefanie M. Schäfer, Zanda Ozoliņa, Zanda Segliņa, Zati Vatansever, Karine Huber

**Affiliations:** 1 ASTRE, Univ Montpellier, Cirad, INRAE, Montpellier, France; 2 Cirad, UMR ASTRE, 34398 Montpellier, France; 3 Cirad, UMR ASTRE, 97170 Petit-Bourg, Guadeloupe France; 4 Institut Agronomique et Vétérinaire Hassan II, Unité Parasitologie et Maladies Parasitaires, 10100 Rabat, Morocco; 5 Institute of Parasitology and Tropical Pathology of Strasbourg, Université de Strasbourg, DIHP UR 7292, 67000 Strasbourg, France; 6 Centre for Monitoring of Vectors, National Reference Centre, Netherlands Food and Consumer Product Safety Authority, Wageningen, The Netherlands; 7 Veterinary Control Central Research Institute, Ankara, Turkey; 8 Department of Parasitology and Parasitic Diseases, Faculty of Veterinary Medicine, Ss. Cyril and Methodius University in Skopje, Skopje, Republic of North Macedonia; 9 Centre for Ecology, Centre for Ecology & Hydrology, Wallingford, OX10 8BB UK; 10 CIISA-Centro de Investigação Interdisciplinar em Sanidade Animal, Faculdade de Medicina Veterinária, Universidade de Lisboa, Avenida da Universidade Técnica, 1300-477 Lisboa, Portugal; 11 Department of Parasitology-Parasitic Diseases, Entomology & Bee Health, Veterinary Centre of Athens, Athens, Greece; 12 Leibniz-Centre for Agricultural Landscape Research, Müncheberg, Germany; 13 Faculty of Agriculture, University of Novi Sad, Novi Sad, Serbia; 14 National Centre for Vector Entomology, Institute of Parasitology, University of Zürich, Zürich, Switzerland; 15 Friedrich-Loeffler-Institut, Federal Research Institute for Animal Health, Greifswald, Germany; 16 Department of Animal Pathology, AgriFood Institute of Aragón (IA2) Veterinary Faculty, 50013 Zaragoza, Spain; 17 Departamento de Microbiología, Laboratorio de Producción y Sanidad Animal de Granada, Junta de Andalucía, Granada, Spain; 18 Doñana Biological Station, CSIC, Sevilla, Spain; 19 Centro de Investigación Biomédica en Red de Epidemiología y Salud Pública (CIBERESP), Madrid, Spain; 20 Centre for Ecology & Hydrology, Edinburgh, OX10 8BB UK; 21 Department of Virology, University of Helsinki, Medicum, Haartmaninkatu 3, Helsinki, 00014 Finland; 22 National Veterinary Research Institute, Puławy, Poland; 23 Istituto Zooprofilattico Sperimentale dell’Abruzzo e del Molise ‘G. Caporale’, Campo Boario, 64100 Teramo, Italy; 24 The Pirbright Institute, Pirbright, UK; 25 Department of Epidemiology and Population Health, Institute of Infection and Global Health, University of Liverpool, Leahurst, Chester High Road, Neston, Cheshire CH64 7TE UK; 26 Applied Zoology and Animal Conservation Research Group, University of the Balearic Islands UIB, Palma, Spain; 27 University of Copenhagen, Copenhagen, Denmark; 28 National Diagnostic and Research Veterinary Medical Institute, Sofia, Bulgaria; 29 Norwegian Veterinary Institute, Oslo, Norway; 30 Institute of Food safety, Animal Health and Environment ‘BIOR’, Riga, Latvia

**Keywords:** *Culicoides* spp., Cryptic species, Phylogeny, Taxonomy, Species delimitation, Palaearctic Region, Biting midge

## Abstract

**Background:**

*Culicoides obsoletus* is an abundant and widely distributed Holarctic biting midge species, involved in the transmission of bluetongue virus (BTV) and Schmallenberg virus (SBV) to wild and domestic ruminants. Females of this vector species are often reported jointly with two morphologically very close species, *C. scoticus* and *C. montanus*, forming the Obsoletus/Scoticus Complex. Recently, cryptic diversity within *C. obsoletus* was reported in geographically distant sites. Clear delineation of species and characterization of genetic variability is mandatory to revise their taxonomic status and assess the vector role of each taxonomic entity. Our objectives were to characterize and map the cryptic diversity within the Obsoletus/Scoticus Complex.

**Methods:**

Portion of the *cox*1 mitochondrial gene of 3763 individuals belonging to the Obsoletus/Scoticus Complex was sequenced. Populations from 20 countries along a Palaearctic Mediterranean transect covering Scandinavia to Canary islands (North to South) and Canary islands to Turkey (West to East) were included. Genetic diversity based on *cox*1 barcoding was supported by *16S* rDNA mitochondrial gene sequences and a gene coding for ribosomal *28S* rDNA. Species delimitation using a multi-marker methodology was used to revise the current taxonomic scheme of the Obsoletus/Scoticus Complex.

**Results:**

Our analysis showed the existence of three phylogenetic clades (*C. obsoletus* clade O2*, C. obsoletus* clade dark and one not yet named and identified) within *C. obsoletus*. These analyses also revealed two intra-specific clades within *C. scoticus* and raised questions about the taxonomic status of *C. montanus*.

**Conclusions:**

To our knowledge, our study provides the first genetic characterization of the Obsoletus/Scoticus Complex on a large geographical scale and allows a revision of the current taxonomic classification for an important group of vector species of livestock viruses in the Palaearctic region.
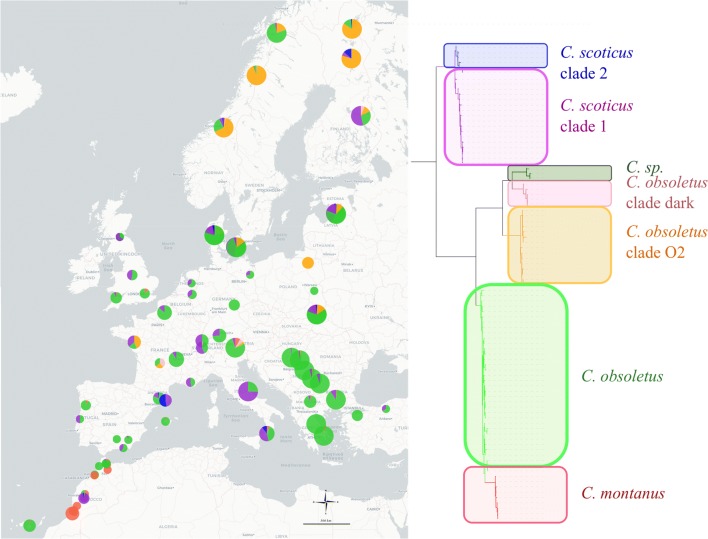

## Background

In 2006, northern Europe faced massive outbreaks of bluetongue disease (BTV), a *Culicoides*-borne viral infection which affects wild and domestic ruminants. This was followed by the emergence of Schmallenberg virus (SBV) in 2011, another *Culicoides*-borne virus, which also caused important economic losses for farmers of domestic ruminants [1]. Both, the emergence and massive spread of these diseases over the Palaearctic region raised questions about the vector competence of native Palaearctic biting midges members of the genus *Culicoides* [2, 3]. Quickly following these epizootics, studies confirmed *Culicoides* species of the subgenus *Avaritia* as the most likely vector species and particularly those of the Obsoletus group (see below) [3–5]. *Culicoides obsoletus*, *C. scoticus*, *C. dewulfi* and *C. chiopterus* are reported to be implicated in BTV and SBV transmission in Europe based on virus detection or isolation in field-collected populations [6]. Laboratory experimental infections have also confirmed the vector competence of *C. obsoletus* and *C. scoticus*, highlighting species variations in their competence level [7].

The literature defines the Obsoletus Group as a group of species with a similar morphology, especially for the characters commonly used for the identification of these insect vectors, namely wing spot pattern (poorly defined spotted wings and a second radial cell with a light spot) and distinctive male genitalia [8–10]. Adults of species in this Group are known to be abundant, widespread across central and northern Europe, and are characterized by long seasonal occurrence [11, 12]. At present, the group is an artificial taxonomic entity still poorly defined, with no real consensus on the included species, with variable internal groupings and naming. Indeed, the world catalogue of *Culicoides* does not account for levels below subgenus and does not identify species complexes as groups [13]. At present, the Obsoletus Group is composed of nine valid species: *C. obsoletus* (Meigen), 1818; *C. sinanoensis* Tokunaga, 1937; *C. scoticus* Downes & Kettle, 1952; *C. montanus* Shakirzjanova, 1962; *C. gornostaevae* Mirzaeva, 1984; *C. abchazicus* Dzhafarov, 1964; *C. filicinus* Gornostaeva & Gachegova, 1972; *C. alachua* Jamnback & Wirth, 1963; and *C. sanguisuga* (Coquillet, 1901). The latter two are the only species belonging to this group exclusively present in the Nearctic region, while the others, are sympatric in the Palaearctic region [8]. *Culicoides obsoletus* is considered Holarctic because it is present in both Nearctic and Palaearctic regions [8]. Combined studies of the geometric morphometry of wings, coupled with molecular analysis have excluded *C. chiopterus* and *C. dewulfi* from the Obsoletus Group, despite previously being considered as part of the Group, based only on morphological features [9, 10, 14–16]. Life-cycles and trophic behaviors for most of the species in the Obsoletus Group are not well described or vary greatly. For instance, ecological niches of *C. chiopterus* and *C. obsoletus* are suspected to be different, although these two species are phylogenetically very close within the subgenus *Avaritia*. *Culicoides obsoletus* is a widespread generalist species and occupies a wide range of larval habitats such as forest litter, silage residue, tree holes or manure [17]. *Culicoides obsoletus* shows opportunistic trophic preferences and is able to take blood meals on various hosts species (man, sheep, goat, cow, horse or rodent) and, occasionally, on birds [18]. *Culicoides chiopterus* is a more specialist species, found engorged almost exclusively on cattle blood with larvae associated with cattle dung [19, 20].

In addition to the group, Meiswinkel et al. [21], defined the Obsoletus Complex to close taxa with very similar female adult morphology, including *C. obsoletus*, *C. montanus* and *C. scoticus*. Several authors have recently reported the existence of cryptic diversity within *C. obsoletus*, namely the *C. obsoletus* clade ‘O2’ and *C. obsoletus* clade ‘O3’ in Sweden and Switzerland [15], and the *C. obsoletus* clade ‘dark’ in the Netherlands [21, 22]. We will use the term “Obsoletus/Scoticus Complex” here to refer to the cryptic species *C. obsoletus*, *C. scoticus* and *C. montanus* as well as all previously described operational taxonomic units in the literature (see above). The terminology “Obsoletus/Scoticus Complex” is written according to the rules defined by Harbach [23] for infrasubgeneric categories within the genus *Anopheles*. Considering that sympatric cryptic species may exhibit different vector competence and may confound epidemiological investigations, it is mandatory to assess the intra- and interspecific diversity within the Obsoletus/Scoticus Complex.

Given the difficulty of separating these species based on morphological identification, various molecular markers have been used to overcome specific identification problems, including *16S* ribosomal DNA [24, 25], *28S* ribosomal DNA [26], cytochrome oxidase *b* (*cytb*), the internal transcribed spacer region 1 (ITS1) [27] and ITS2 rDNA [28]. However, the DNA region primarily used to infer phylogenetic relationships in this complex has been the mitochondrial cytochrome *c* oxidase subunit 1 (*cox*1) [29]. Most of the diversity within the Obsoletus/Scoticus Complex has been identified using *cox*1 [29–33]. Despite the diversity of markers used to characterize the phylogeny of the complex, few studies have used a multi-marker approach [34]. This study, therefore, will integrate a multi-marker approach in order to strengthen genetic reconstruction of the Obsoletus/Scoticus Complex.

The Obsoletus/Scoticus Complex, as currently presented in the literature, is confused and needs taxonomic revision. We characterized and mapped the genetic diversity of the Obsoletus/Scoticus Complex along a Palaearctic-Mediterranean transect covering Scandinavia to Canary islands (North to South) and Canary islands to Turkey (West to East). Our main objectives were to identify and describe the cryptic diversity observed within the Obsoletus/Scoticus Complex over a wide geographical area using molecular analyses and to question the taxonomic status of some newly described clades. In order to achieve these objectives, we conducted a molecular analysis which combined multi-marker sequencing, phylogenetic analyses and species delimitation to explore the genetic diversity of the Obsoletus/Scoticus Complex in the western European portion of the Palaearctic region.

## Methods

### *Culicoides* capture and morphological identification

Biting midges were collected at 68 sites located in 20 countries in the western European portion of the Palaearctic region, between 2009 and 2017, using national surveillance networks for *Culicoides* populations or local collections (Additional file 1: Table S1). Collections were made overnight with Onderstepoort Veterinary Institute (OVI) light traps set at farms near horses, cattle or sheep and all insects were stored in 70% ethanol. Morphological identification to the species level of adult *Culicoides* spp. was performed under a binocular microscope using the available identification keys [35, 36].

### DNA extraction, amplification and sequencing

DNA was extracted from a total of 3883 adult females belonging to the Obsoletus/Scoticus Complex using the NucleoSpin® DNA kit RapidLyse (Macherey-Nagel, Duren, Germany), following the manufacturer’s instructions. An additional step was added, before extraction, for all individuals (specimens were ground in 50 μl of 1× PBS buffer). DNA samples are available upon request. Fragments of *cox*1 were amplified for the 3883 individuals. After sequence cleaning, 3763 sequences of *cox*1 were obtained [dataset *cox*1 (1)].Fragments of *16S* and *28S* rDNA were amplified on individuals chosen to be representative of the entire species diversity resulting from *cox*1 [dataset *cox*1 (2)] to reinforce mitochondrial gene sequences. All primer sequences as well as the information relating to them are present in Additional file 2: Table S2. PCR’s were performed in a 25 μl reaction volume. The PCR mix contained 1× Qiagen buffer, 1 mM MgCl_2_, 0.25 mM of each dNTP, 0.2 μM of each primer, 1.25 U Qiagen Taq Polymerase and 0.7 ng/μl genomic DNA for all genes. PCR programs included one-step of 5 cycles before a second step with 35 cycles for *16S* rDNA and *28S* rDNA. PCR amplification conditions were: an initial denaturation step at 94 °C for 5 min followed by 5 cycles of 94 °C for 30 s; 45 °C for *cox*1, 42 °C for *16S* rDNA or 55 °C for *28S* rDNA for 40 s; 72 °C for 1min; 35 cycles of 94 °C for 30 s; 51 °C for *cox*1, 55 °C for *16S* rDNA or 50 °C for *28S* rDNA for 30 s; 72 °C for 1 min; and a final extension step at 72 °C for 10 min. For each amplification reaction, negative controls were carried out. The PCR products were visualized on 1.5% agarose gels with a GelRed® Nucleic Acid Gel Stain, staining after migration of 90 min at 130 V by electrophoresis for quality control. After purifications, carried out by the sequencing service provider, the remaining 20 μl were sequenced with the same forward primers used for PCR (https://www.genewiz.com).

### Sequence analyses

The reference sequences of *cox*1 used to identify individuals to species are available in Additional file 3: Table S3. A total of 3763 *cox*1 sequences (Additional file 4: Table S4) from female adults morphologically identified as belonging to the Obsoletus/Scoticus Complex were obtained after deletion of short and poor-quality sequences. The *cox*1 alignment was used to identify all *Culicoides* to species- or clade-level within the complex using the reference sequences. Among the reference sequences used to specifically assign our *Culicoides* samples, some sequences previously identified as *C. obsoletus* O1 and O3 [15] were included in our analysis in order to cover the diversity of the clades described in the literature within the Obsoletus/Scoticus Complex. After comparison with other sequences, it appears that the sequences named *C. obsoletus* O1 were actually *C. obsoletus* and that *C. obsoletus* O3 belongs to *C. obsoletus* clade dark. For this purpose, *cox*1 sequences were aligned with reference sequences [dataset *cox*1 (1)]. A phylogenetic tree based on maximum likelihood method allowed designation of a species name to each sequence if the latter belonged to a monophyletic clade with strong support (bootstrap < 900) that included a reference sequence. Thus, *Culicoides* were sequenced for markers *16S* rDNA and *28S* rDNA to support the phylogenetic reconstruction of the complex. All *cox*1 [dataset *cox*1 (2)], *16S* rDNA and *28S* rDNA sequences were independently aligned with the MUSCLE [37] algorithm available in the software GENEIOUS v.6.0.5 (Biomatters, http://www.geneious.com). Genetic diversity indices, haplotype and nucleotide diversity were evaluated using DNASP v.5.10 [38]. Alignments with gaps were cleaned using the software Gblocks 0.91b [39]. To assess genetic distance between clades and species within, barcoding gap bar chart using R software was performed with *ggplot2* [40] and *ggthemes* packages. Intra- and interspecific genetic differences based on the Kimura 2-Parameter (K2P) distance model [41, 42] were calculated with MEGAX [43]. In order to map the specific diversity of the Obsoletus Group, the R software version 3.6.0 was used with the *Leaflet* version 2.0.2 and *shiny* packages version 1.4.0.

### Phylogenetic inferences

Phylogenetic trees were constructed for the three markers using maximum-likelihood (ML) and Bayesian inference (BI). Bayesian inference analyses were conducted on MrBayes version 3.2.6, with tree sampling every 1000 generations in order to calculate posterior probabilities (PP) and 10 million generations. Optimal sequence evolutionary models for each analysis were obtained with Bayesian information criterion (BIC) using jModelTest. Maximum-likelihood analyses were conducted on PhyML 3.0. The ML analyses were conducted with the best model selected using 1000 bootstrap replicates for each dataset to investigate the level of support at each node, with starting tree determined by BioNJ analysis. After independent analysis of each gene, alignment of *cox*1 and *16S* rDNA were concatenated, and analyzed following ML and BI methods.

### Species delimitation methods

Two species delimitation methods were applied. The first method was a Bayesian implementation of classical GMYC method, Bayesian General Mixed Yule Coalescent (bGMYC) [44]. The single-locus ultrametric gene trees used for bGMYC methods were created with BEAST 1.8.0 [45] under a strict clock model, a Yule Process Tree Model of speciation, and a random starting tree. This analysis was carried out with default prior distribution, without outgroups and with 10 million generations sampled every 1000 cycles with HKY + G substitution model for *16S* rDNA, and with HKY + I for *28S* rDNA [46]. The software TREEANNOTATOR v1.8.2 was used to find Ultrametric maximum clade credibility (MCC). Single-threshold GMYC analyses were conducted with *splits* package in R.

The second species delimitation method used was the Bayesian Poisson Tree Processes (bPTP) through the web server PTP (http://species.h-its.org/ptp/) [47] with 100,000 MCMC generations and a thinning parameter of 100 on a maximum likelihood phylogenetic tree constructed with *cox*1 and *16S* rDNA genes concatenated with *C. dewulfi* as outgroup.

## Results

### Molecular analysis

In total, 3763 sequences were obtained for *cox*1, 95 for *16S* rDNA and 95 for *28S* rDNA (Table 1). All sequences were deposited in GenBank (Additional file 4: Table S4). No stop codons, insertions or deletions were found in any of the *cox*1 sequences, indicating functional mitochondrial products.

**Table 1:** Sequence statistics for four gene fragments used to reconstruct the phylogeny of the Obsoletus/Scoticus complex

Dataset	*n*	Length (bp)	S	C+G (%)	h	Hd (SD)	π (SD)	Nucleotide model (under BIC)	Implemented model (BI)
*cox*1 (1)	3763	512–627	141	34.5	228	0.890 (0.003)	0.06299 (0.00473)		
*cox*1 (2)	95	528–623	146	33.7	77	0.994 (0.003)	0.0921 (0.00489)	TPM2uf+I+G	nst = 6; rates = invgamma
*16S* rDNA	95	263	51	15.8	13	0.851 (0.02)	0.04791 (0.00446)	HKY+G	nst = 2; rates = gamma
*28S* rDNA	95	576	25	39.8	24	0.911 (0.015)	0.00753 (0.00099)	HKY+I	nst = 2; rates = propinv
Concatened genes	95	731	197	32.8	90	0.998 (0.002)	0.04594 (0.00297)	TPM2uf+I+G	nst = 6; rates = invgamma

*Abbreviations*: n, number of individuals; h, number of haplotypes; Hd, haplotype (gene) diversity; π, nucleotide diversity; S, number of polymorphic sites; SD, standard deviation; bp, base pairs; BIC, Bayesian information criterion; BI, Bayesian inference

**Fig. 1:**
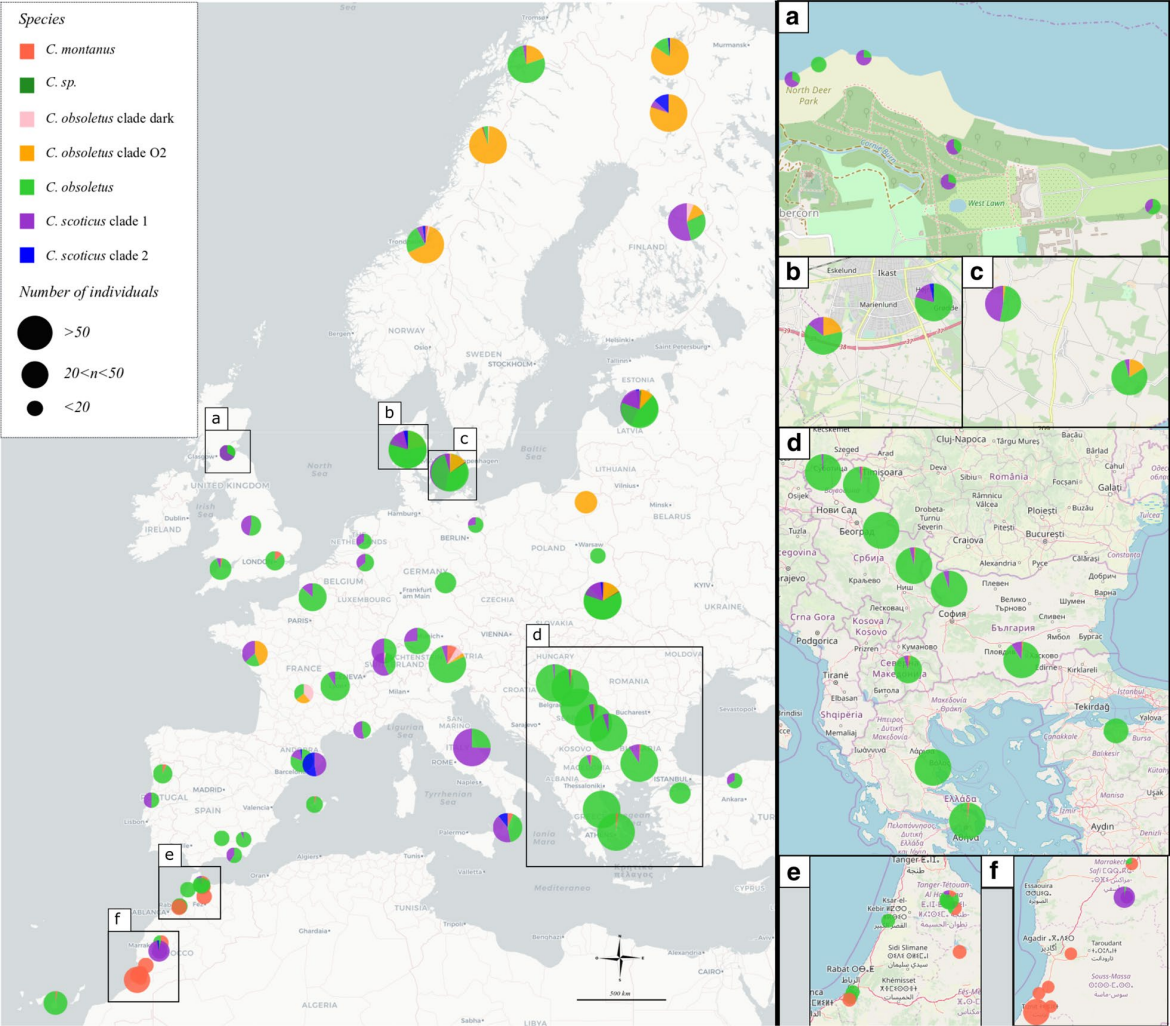
Map of biting midges sampling sites represented by the number of individuals per clade within the Obsoletus/Scoticus Complex. The different clades identified are shown in different colours. The size of circles on Europe map correspond to the number of individuals per clade within the cryptic species. The numbered maps on the right correspond to magnifications of some study areas. The sites of sampling too close and thus the pie charts this superimposed are to be taken into account in the numbered magnifications and not on the main map. Magnifications: **a** Scotland sample sites; **b**, **c** Denmark sample sites; **d** Balkans sample sites; **e**, **f** Morroco sample sites

Within the selected samples present in our data set, the most abundant species was *C. obsoletus*, with 2416 individuals sampled, representing 68% of all *Culicoides* caught (Fig. 1). Species diversity within the complex varied according to the latitudes of the sampling sites (Fig. 1). The most sampled species in northern Europe (Norway and Finland) was the *C. obsoletus* clade O2 with 62% (162 individuals) and 58% (147 individuals) of this clade, respectively, in each country. For eastern Europe (Latvia, Poland, Serbia, Bulgaria, Macedonia, Greece and Turkey) the most sampled species was *C. obsoletus*, representing, for example, 99% (162 individuals) of all *Culicoides* sampled in Greece. However, a population in Poland (Wronka) appears to be an exception with 100% (35 individuals) of *C. obsoletus* clade O2. Three individuals, belonging to a phylogenetic clade unidentifiable by our reference sequences close to *C. obsoletus* dark, were reported from Latvia. The latter sequences are identical to sequences present in the BOLD database (accession numbers: GMGRC1056-13, GMGRC1000-13, GMGRD2587-13) of *Culicoides* collected in Bavaria, Germany. Finally, western and central Europe (Portugal, Spain, UK, France, Italy, Netherlands, Germany, Switzerland and Denmark) had the higher species diversity of all the species found in the Palaearctic transect. The most sampled species within this area was *C. obsoletus* with 913 individuals, representing 64% of the *Culicoides* sampled. However, unlike in eastern Europe, *C. scoticus* clade 1 was also found in significant numbers with 386 individuals, or 27% of the samples. *Culicoides obsoletus* clade dark was rarely reported in Europe with only 26 individuals found in France, Denmark, Finland, Italy, Latvia, Norway and Switzerland. *Culicoides montanus* was found in relatively high proportion in Morocco with 54% (80 individuals) of samples, whereas it was much rarer and more sporadic in European countries.

**Fig. 2:**
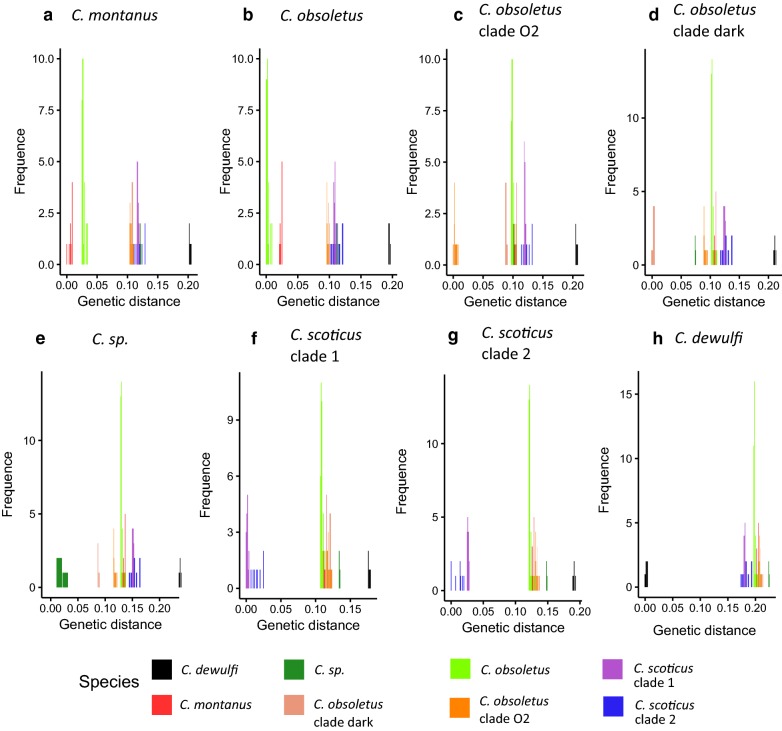
Relative distribution of interspecific divergence and intraspecific variation in *cox*1 for all cryptic species within the Obsoletus/Scoticus Complex

A total of 228 different *cox*1 haplotypes were identified. Haplotype diversity varied from 0.829 for *C. obsoletus* clade O2 to 0.517 for *C. scoticus* clade 1 and *C. obsoletus* clade dark (Table 2). In accordance with mitochondrial genes in insects [48], the GC composition ranged from 16% for *16S* rDNA alignment to 39.8% for *28S* rDNA, with a strong AT bias (Table 2). *Culicoides obsoletus* showed very high intraspecific diversity with 94 different haplotypes, representing nearly 43% of the total haplotypic diversity encountered in this study. The maximum interspecific genetic distance (Fig. 2) was reached between *C. dewulfi* and other taxonomic units, with a minimum of 17% of genetic distance between this outgroup and all other members inside the complex. *Culicoides dewulfi* is used here as an outgroup. Genetic distances of the same level as the other intraspecific distances were observed between *C. scoticus* clade 1 and *C. scoticus* clade 2, and between *C. obsoletus* and *C. montanus*, with a maximum of 2% and 4%, respectively. Similar interspecific genetic distances, were observed between all the other clades within the complex, with a minimum of 8% distance. All species had mean intraspecific distances of less than 1%, other than *C. scoticus* clade 2.

**Table 2:** Genetic diversity indices for mitochondrial *cox*1 gene segment of *Culicoides* spp. in the Obsoletus/Scoticus Complex

Species	*n*	h	Hd (SD)	π (SD)	S	C+G (%)
*C. montanus*	106	13	0.642 (0.039)	0.00726 (0.00317)	66	0.334
*C. obsoletus*	2416	106	0.773 (0.005)	0.00450 (nd)	72	0.324
*C. obsoletus* clade O2	512	38	0.829 (0.012)	0.00447 (nd)	53	0.310
*C. obsoletus* clade dark	26	6	0.517 (0.113)	0.00191 (0.00058)	7	0.315
*Culicoides* sp.	3	2	0.667 (0.314)	0.00749 (0.00353)	6	0.331
*C. scoticus* clade 1	645	61	0.562 (0.023)	0.00463 (nd)	93	0.347
*C. scoticus* clade 2	55	12	0.753 (0.046)	0.00499 (0.00093)	16	0.328

*Abbreviations*: n, number of individuals; h, number of haplotypes; Hd, haplotype (gene) diversity; π, nucleotide diversity; nd, not determined; S, number of polymorphic sites; SD, standard deviation

Among the 228 unique *cox*1 haplotypes, 95 were selected to represent the specific diversity of the complex. Using the same individuals, two alignments of 95 sequences of *16S* rDNA (Additional file 5: Figure S1.) and *28S* rDNA were constructed. In order to compare tree topology and to concatenate markers, a second *cox*1 alignment of the 95 sequences was performed (Additional file 6: Figure S2.). The *cox*1 dataset had a greater haplotype diversity than *16S* rDNA and *28S* rDNA datasets, with 0.994 *vs* 0.851 and 0.911 respectively. *28S* rDNA was more monomorphic than *cox*1 and *16S* rDNA, with 25, 146 and 51 polymorphic sites, respectively.

### Phylogenetic analysis

**Fig. 3:**
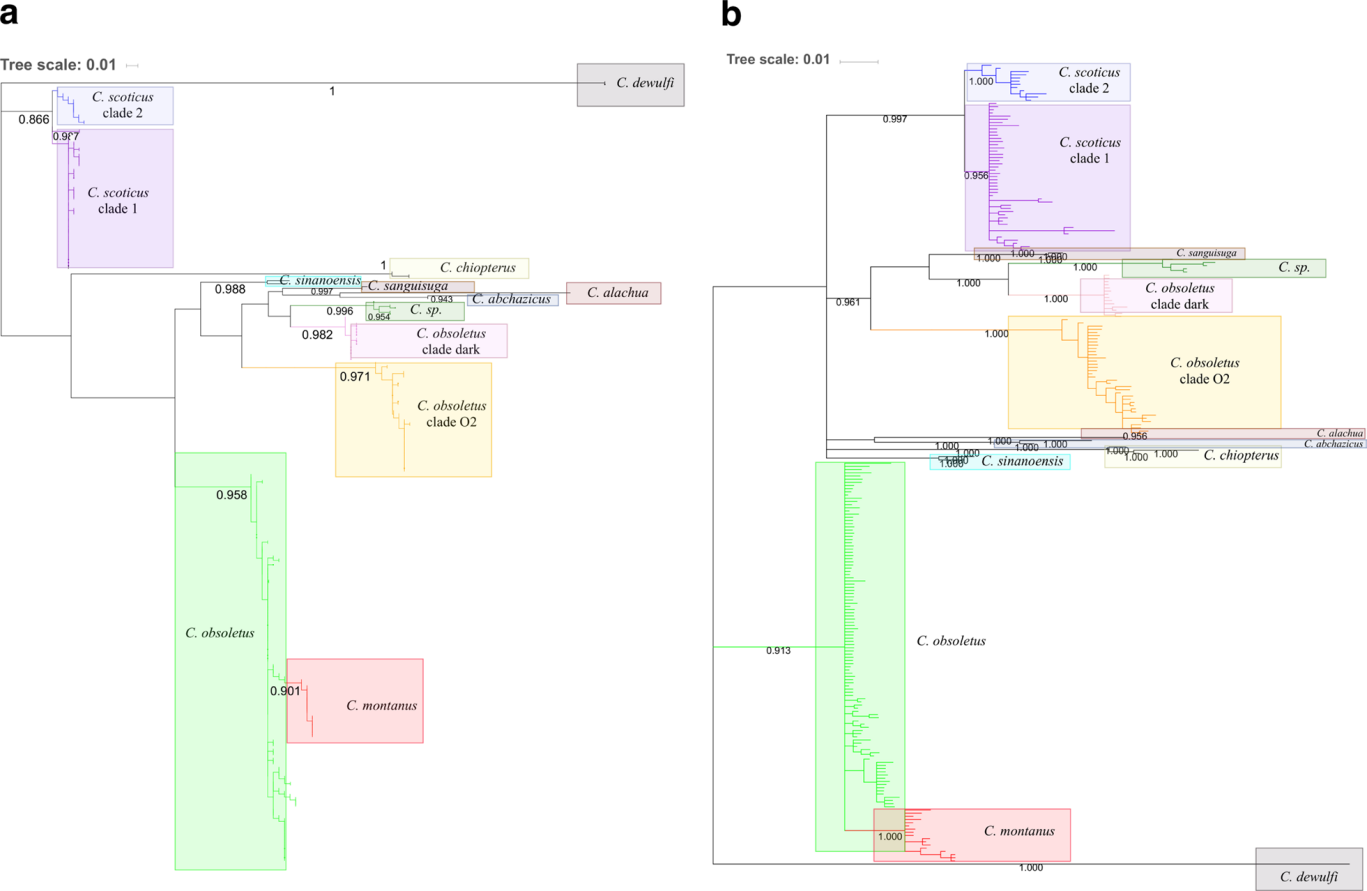
Maximum likelihood (**a**) and Bayesian inference (**b**) phylogenetic tree using *cox*1 representing the haplotypic diversity within the Obsoletus/Scoticus Complex at the European scale. Values at the nodes represent bootstrap (**a**) and posterior probability (**b**) values greater than 900 for the most important nodes (1000 replicates)

Information on the alignments used for the construction of phylogenetic trees is provided in Table 1. Species with confirmed taxonomic validity (*C. montanus*, *C. obsoletus* and *C. scoticus*) and cryptic taxa (*C. obsoletus* clade O2, *C. obsoletus* clade dark) highlighted by previous studies were strongly supported (bootstrap > 90%) (Fig. 3). *Culicoides obsoletus* clade O2 constituted a monophyletic clade with strong support (bootstrap > 90%). *Culicoides obsoletus* and *C. montanus* formed a monophyletic clade. C*ulicoides scoticus*, another species considered valid, showed two phylogenetic clades, i.e. *C. scoticus* clade 1 and *C. scoticus* clade 2. A monophyletic clade close to *C. obsoletus* clade dark, was strongly supported by bootstrap values. Topologies of phylogenetics trees constructed *via* maximum likelihood (Fig. 3a) and Bayesian inference (BI) analyses (Fig. 3b) were congruent for alignment of full haplotype diversity.

### Species delimitation

**Fig. 4:**
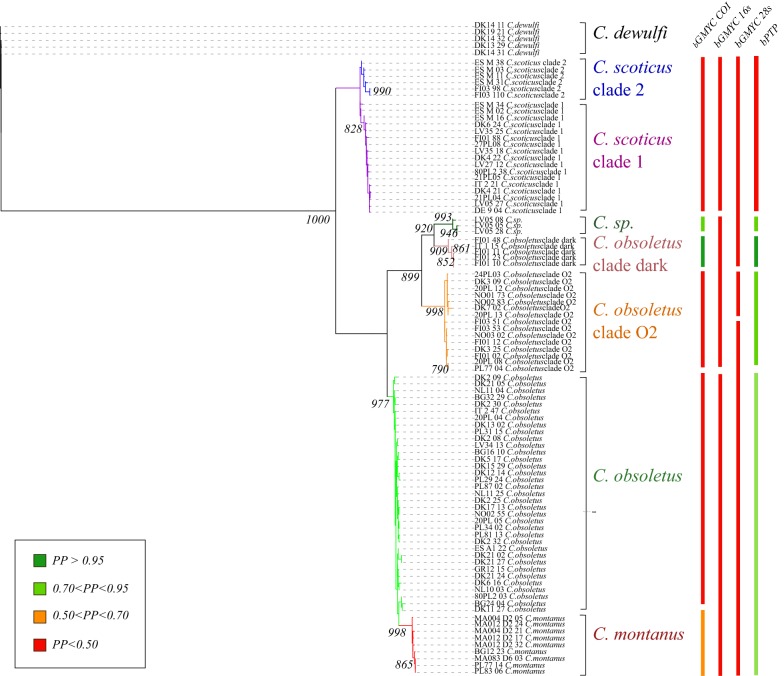
Maximum likelihood phylogenetic tree using concatenated genes (*cox*1; *16S* rDNA) representing species delimitation and molecular relationships within the Obsoletus/Scoticus Complex

Using the *cox*1 dataset and bGMYC method for species delimitation (Fig. 4), 6 molecular operational taxonomic units (MOTUs) were observed: *C. montanus*, C*. obsoletus*, *C. scoticus*, *C. obsoletus* clade O2, *C. obsoletus* clade dark and *Culicoides* sp. within the Obsoletus/Scoticus Complex. Based on the *16S* rDNA dataset analysed with the same delimitation method, three MOTUs were characterised including (i) two clades within *C. scoticus*; (ii) *Culicoides* sp., C. *obsoletus* clade dark and C. *obsoletus* clade O2; and (iii) *C. obsoletus* and *C. montanus*. Species delimitation with the *28S* rDNA and *16S* rDNA dataset had a lower resolution compared to *cox*1. The *28S* rDNA and *16S* rDNA datasets were invaluable in identifying cryptic diversity within the Obsoletus/Scoticus Complex. Indeed, the tree generated with the *28S* rDNA dataset showed very low polymorphisms and resolution signal (Additional file 7: Figure S3.). The bPTP method was conducted on concatenated dataset of *cox*1 and *16S* rDNA genes. The best statistical support for species delimitation was for concatenated dataset with bPTP. MOTUs found with the concatenated alignment and *cox*1 were the same, except for the delimitation of *C. montanus* and *C. obsoletus*. Indeed, using molecular delineation based on bPTP analysis, MOTUs were observed for *C. scoticus*, *C. obsoletus* clade O2, C. *obsoletus* clade dark, and *Culicoides* sp. Using the bPTP method we were not able to distinguish *C. obsoletus* and *C. montanus* as well as *C. scoticus* clades 1 and 2, which were not classified as separate species by either method.

## Discussion

Our study investigated the genetic diversity of the Obsoletus/Scoticus Complex at the European level (68 sampling sites across 20 Palaearctic countries with 3763 *cox*1 sequences). For the first time, the sample selection covers the whole known western Palaearctic distribution area of these species. The complementary use of the mitochondrial *16S* rDNA and nuclear *28S* rDNA genes confirms the important level of cryptic diversity found within the Obsoletus/Scoticus Complex. Indeed species delimitation methods allowed us to delineate five MOTUs and: (i) to provide evidence of the taxonomic validity of *C. obsoletus* clade O2 and *C. obsoletus* clade dark (ii) to identify individuals belonging to a species not yet described or not present into the databases; and (iii) to question the taxonomic status of *C. montanus.*

Species assignment at the European scale showed variation in the distribution of the cryptic diversity of Obsoletus/Scoticus Complex. This result confirmed a previous study by Möhlmann et al. [49], who found a strong latitudinal effect on the relative abundance of species of the Obsoletus/Scoticus Complex. However, the previous study was carried out with few individuals from a relatively small number of countries and sampling sites [49]. This contrasts with our study, the first to be conducted at a European scale with a large data set sufficient to provide a more precise idea of the cryptic diversity within the Obsoletus/Scoticus Complex. The latitudinal variation in the relative abundance of the different cryptic species in the Obsoletus/Scoticus Complex, could be due to a wide range of factors like different ecological niches, or differences in the availability of hosts and breeding sites [50]. For example, a study conducted in Italy showed that *C. scoticus* collection sites were dominated by areas of natural vegetation or forest, at medium altitudes, preferably in wilder and more pristine environments [51]. However, the heterogeneity of *Culicoides* collection dates may also explain these variations in specific diversity within the complex. For instance, a study conducted in Sweden, found a seasonal variation in *Culicoides* community structure [52]. The omnipresence of *C. obsoletus* makes it the dominant species in Europe, confirming its status as a generalist species, which tolerates a wide range of eco-climatic conditions. The dominant species also varies according to geographical location. For example, *C. obsoletus* clade O2 is the most sampled species in Nordic countries while *C. montanus* prevails in Morocco. France, Italy and Spain appear to have the highest specific diversity of *Culicoides* belonging to the Obsoletus/Scoticus Complex. These three countries bring together all the cryptic diversity known so far, except the new, unidentified, *Culicoides* taxon that has only been found in Latvia regarding our dataset. This could be due to the significant diversity of ecological niches as well as the high density of hosts in these countries and this species diversity variation is in line with general patterns of latitudinal increase in species richness [53].

Our phylogenetic analysis allowed us to define seven well supported phylogenetic clades. Some of them correspond to species with taxonomic validity (*C. montanus*, *C. obsoletus* and *C. scoticus*), some more recently described phylogenetic clades (*C. obsoletus* clade dark and *C. obsoletus* clade O2) [52, 54] and some clades never described before (*Culicoides* sp. and *C. scoticus* clade 2).

Without taking into account *C. scoticus*, the phylogenetic reconstruction produced herein, confirmed the presence of two divergent groups; one consisting of C. *obsoletus* and *C. montanus* and the other of *C. obsoletus* clade dark, C. *obsoletus* clade O2 and a clade not yet described in the literature. We were unable to identify these sequences due to the absence of reference sequences identified at the species level in the sequence databases. However, three sequences, from specimens collected in Bavaria in Germany, present in the BOLD database are identical. Further sampling and sequencing of *Culicoides* from eastern Europe are necessary in order to associate morphological features with this new cryptic species. The large number of *Culicoides* processed during this study made it difficult to use non-destructive DNA extraction techniques. Non-destructive techniques would be necessary to couple morphological criteria with genetic analysis, in order to identify this species.

We also described a second clade phylogenetically very close to *C. scoticus*, C. *scoticus* clade 2 [55]. However, given the small genetic distance observed between these two clades, they can be considered as intraspecific variation within *C. scoticus*.

According to this study, *C. obsoletus* clade dark appears as a true cryptic species, with high phylogenetic support. Meiswinkel et al. [21] hypothesized that *C. obsoletus* clade dark could be *C. gornostaevae* Mirzaeva, 1984, but *C. gornostaevae* was significantly larger and had a distribution restricted to the boreal zone of Siberia. However, this species has been recently reported from Norway, Poland and Sweden [56] but the lack of *C. gornostaevae* reference sequences in publicly available databases prevented comparisons with *C. obsoletus* clade dark.

*Culicoides obsoletus* clade O2 was also strongly supported by phylogenetic and species delimitation analysis, and thus could be considered a cryptic species within the Obsoletus/Scoticus Complex. However, our sample showed a high abundance of *C. obsoletus* clade O2 mainly at high latitudes, whereas it had initially been identified for the first time further south in the Swiss Alps [54, 56, 57] and France.

Little is known about the ecology of *C. montanus*. We found this species over a large geographical area, from Morocco to a few individuals in Norway. In spite of this, we have identified only a very small number of *C. montanus* except in Morocco where this species constitutes the majority. This latter result is in accordance with the fact that sites in Italy, where *C. montanus* is predominant are characterised by a high land surface temperature, higher than the values registered in the *C. obsoletus* and *C. scoticus* sites [51]. *Culicoides obsoletus* and *C. scoticus* are considered to be sibling species because of diagnostic female morphological characters, which are difficult to observe or overlap [16], as opposed to the morphological identification of males which is simpler [58]. If the genetic analyses by Pages & Sarto [58] confirm that both *C. obsoletus* and *C. scoticus* are distinct species, the question remains open for *C. montanus.* Previous phylogenetic studies based on *cox*1 indeed showed limited genetic distance between *C. obsoletus* and *C. montanus* and in studies based on ITS sequences *C. montanus* always appears in one of the subclades of *C. obsoletus* [59]. In our multi-marker phylogenetic tree, *C. montanus* and *C. obsoletus* formed a monophyletic clade. In addition, the genetic distance between *C. obsoletus* and *C. montanus* was of the same order of magnitude as some intraspecific distances.

The number of putative species defined within the Obsoletus/Scoticus Complex varied depending on the molecular markers and species delimitation methods used (Fig. 4), particularly pertaining to the status of *C. obsoletus* and *C. montanus*. Unlike the other methods, the bGMYc method, based on *cox*1, distinguished these two species. This can be explained by the fact that some parameters of the analysis (i.e. priors), like differences in population size or speciation rates, can bias the GMYC method by overestimating the number of species [46, 60–62]. Insufficient sampling, high gene flow or a recent speciation event are also likely explanations for the differences in results between phylogenetic trees and species delimitation results [63, 64]. Although subject to the same constraints, it has been shown that the bPTP method is significantly more robust [65]. Moreover, methods based on multiple loci improve discovery, resolution and stability of species delimitation [66, 67]. Furthermore, studies have shown that species delineation in insects is more appropriate with multilocus species delimitation methods [68–71]. These arguments allowed us to validate the species delimitation scheme produced with the bPTP method. This analysis coupled with the low level of genetic distances observed between *C. montanus* and *C. obsoletus* led us to question the taxonomic status of *C. montanus*. This could be the beginning of a speciation process of *C. montanus* within the clade of *C. obsoletus.* Hovewer, *C. montanus* was originally described from Kazakhstan and present in central Asia [57]. Therefore, examination of individuals sampled in this geographical area could strengthen our conclusions.

In the light of our conclusions, in-depth morphological analyses with the deposition of reference individuals will have to be carried out in order to decide whether or not to definitively rule out the taxonomic status of the cryptic species making up this complex. Indeed, although adult females are not morphologically distinguishable on a routine basis, males are easier to identify using their genitalia and pupal differences and can provide evidence of morphological differences between the species.

## Conclusions

This study provides clarification of the distribution pattern of species belonging to the Obsoletus/Scoticus Complex, using a dataset based on samples from western Palaeartic and Mediterranean transect. Strong variations in latitudinal cryptic species diversity was observed. This study clarifies the phylogenetic relationships between species belonging to the Obsoletus/Scoticus Complex. We identified and validated five MOTUs, *C. obsoletus*, *C. scoticus*, *C. obsoletus* clade O2, *C. obsoletus* clade dark and a MOTUs corresponding to an unidentified species. The latter three species have not been formally described but our results confirm that they should be considered as species in their own right. More detailed studies of their morphology and ecology are needed to provide more detailed descriptions of these species. Furthermore, our results raise questions concerning the taxonomic status of *C. montanus*, which was previously considered as a taxonomically valid species.

## Supplementary information

**Additional file 1: Table S1.** Information on adult female *Culicoides* sampling sites and results of specific assignation using *cox*1.

**Additional file 2: Table S2** Primers used for PCRs and sequencing in this study.

**Additional file 3: Table S3.** Reference sequences used for specific assignation.

**Additional file 4: Table S4.** Information on all *Culicoides* sequenced and GenBank accession numbers.

**Additional file 5: Figure S1.** Maximum likelihood phylogenetic tree using *16S* rDNA. Values at the nodes represent bootstrap values greater than 800 for the most important nodes (1000 replicates).

**Additional file 6: Figure S2.** Maximum likelihood phylogenetic tree using *cox*1. Values at the nodes represent bootstrap values greater than 800 for the most important nodes (1000 replicates).

**Additional file 7: Figure S3.** Maximum likelihood phylogenetic tree using *28S* rDNA. Values at the nodes represent bootstrap values greater than 800 for the most important nodes (1000 replicates).

## Data Availability

All data generated or analysed during this study are included in this published article and its additional files. The newly generated sequences were submitted in the GenBank database under the accession numbers MT170026-MT173788.

## Abbreviations

BTV: bluetongue virus
SBV: Schmallenberg virus
*cytb*: cytochrome oxidase b
ITS1: internal transcribed spacer region 1
ITS2: internal transcribed spacer region 2
*cox*1: cytochrome *c* oxidase subunit 1
N: number of individuals
h: number of haplotypes
Hd: haplotype (gene) diversity
π: nucleotide diversity
nd: not determined
S: number of polymorphic sites
bGMYC: Bayesian General Mixed Yule Coalescent
MOTUs: molecular operational taxonomic units
bPTP: Bayesian Poisson Tree Processes
