# Correction to: The tree that hides the forest: cryptic diversity and phylogenetic relationships in the Palaearctic vector Obsoletus/Scoticus complex (Diptera: Ceratopogonidae) at the European level

**DOI:** 10.1186/s13071-020-04349-y

**Published:** 2020-09-22

**Authors:** Antoine Mignotte, Claire Garros, Laetitia Gardès, Thomas Balenghien, Maxime Duhayon, Ignace Rakotoarivony, Laura Tabourin, Léa Poujol, Bruno Mathieu, Adolfo Ibañez-Justicia, Ahmet Deniz, Aleksandar Cvetkovikj, Bethan V. Purse, David W. Ramilo, Despoina Stougiou, Doreen Werner, Dubravka Pudar, Dušan Petrić, Eva Veronesi, Frans Jacobs, Helge Kampen, Isabel Pereira da Fonseca, Javier Lucientes, Javier Navarro, Josue Martinez-de la Puente, Jovana Stefanovska, Kate R. Searle, Khalid Khallaayoune, C. Lorna Culverwell, Magdalena Larska, Maria Bourquia, Maria Goffredo, Marina Bisia, Marion England, Matthew Robin, Michela Quaglia, Miguel Ángel Miranda-Chueca, René Bødker, Rosa Estrada-Peña, Simon Carpenter, Simona Tchakarova, Sofia Boutsini, Ståle Sviland, Stefanie M. Schäfer, Zanda Ozoliņa, Zanda Segliņa, Zati Vatansever, Karine Huber

**Affiliations:** 1 ASTRE, Univ Montpellier, Cirad, INRAE, Montpellier, France; 2 Cirad, UMR ASTRE, Montpellier, F-34398 France; 3 Cirad, UMR ASTRE, Petit-Bourg, F- 97170 Guadeloupe, France; 4 Unité Parasitologie et Maladies Parasitaires, Institut Agronomique et Vétérinaire Hassan II, Rabat, 10100 Morocco; 5 Institute of Parasitology and Tropical Pathology of Strasbourg, Université de Strasbourg, DIHP UR 7292, Strasbourg, F-67000 France; 6 Centre for Monitoring of Vectors, National Reference Centre, Netherlands Food and Consumer Product Safety Authority, Wageningen, The Netherlands; 7 Veterinary Control Central Research Institute, Ankara, Turkey; 8 Department of Parasitology and Parasitic Diseases, Faculty of Veterinary Medicine, Ss. Cyril and Methodius University, Skopje, Republic of North Macedonia; 9 Centre for Ecology and Hydrology, UK Centre for Ecology and Hydrology, Wallingford, OX10 8BB UK; 10 CIISA - Centro de Investigação Interdisciplinar em Sanidade Animal, Faculdade de Medicina Veterinária, Universidade de Lisboa, Avenida da Universidade Técnica, Lisboa, 1300-477 Portugal; 11 Veterinary Centre of Athens Department of Parasitology-Parasitic Diseases, Entomology & Bee Health, Athens, Greece; 12 Leibniz-Centre for Agricultural Landscape Research, Müncheberg, Germany; 13 Faculty of Agriculture, University of Novi Sad, Novi Sad, Serbia; 14 National Centre for Vector Entomology, Institute of Parasitology, University of Zürich, Zürich, Switzerland; 15 Friedrich-Loeffler-Institut, Federal Research Institute for Animal Health, Greifswald, Germany; 16 Department of Animal Pathology, AgriFood Institute of Aragón (IA2) Veterinary Faculty, Zaragoza, 50013 Spain; 17 Laboratorio de Producción y Sanidad Animal de Granada, Departamento de Microbiología, Junta de Andalucía, Granada, Spain; 18 Doñana Biological Station, CSIC, Sevilla, Spain; 19 Centro de Investigación Biomédica en Red de Epidemiología y Salud Pública (CIBERESP), Madrid, Spain; 20 Centre for Ecology & Hydrology, Edinburgh, OX10 8BB UK; 21 Department of Virology, University of Helsinki, Medicum, Haartmaninkatu 3, Helsinki, 00014 Finland; 22 National Veterinary Research Institute, Puławy, Poland; 23 Istituto Zooprofilattico Sperimentale dell’Abruzzo e del Molise ‘G. Caporale’, Campo Boario, 64100 Teramo, Italy; 24 The Pirbright Institute, Pirbright, UK; 25 Department of Epidemiology and Population Health, Institute of Infection and Global Health, University of Liverpool, Leahurst, Chester High Road, Neston, Cheshire, Leahurst, CH64 7TE UK; 26 Applied Zoology and Animal Conservation Research Group, University of the Balearic Islands UIB, Palma, Spain; 27 University of Copenhagen, Copenhagen, Denmark; 28 National Diagnostic and Research Veterinary Medical Institute, Sofia, Bulgaria; 29 Norwegian Veterinary Institute, Oslo, Norway; 30 Institute of Food safety, Animal Health and Environment ‘BIOR’, Riga, Latvia; 31 Faculty of Veterinary Medicine, Department of Parasitology, Kafkas University, Kars, Turkey

## Correction to: Parasites Vectors (2020) 13:265 10.1186/s13071-020-04114-1

Following publication of the original article [1], the authors flagged that unfortunately there is an error with the affiliations.

The affiliation of Zati Vatansever (31) is listed as “Veterinary Control Central Research Institute, Ankara, Turkey”.

However, the correct affiliation is “Faculty of Veterinary Medicine, Department of Parasitology, Kafkas University, Kars, Turkey”.

The authors apologize for the inconvenience caused.
